# Anti-synthase syndrome associated with SARS-Cov-2 infection

**DOI:** 10.1186/s12890-024-02966-2

**Published:** 2024-04-15

**Authors:** Xing-Yue Chen, Jun Chen, Li-Jia Zhi, Kun-Lan Long, Pei-Yang Gao

**Affiliations:** Hospital of Chengdu University of Traditional Chinese Medicine, Chengdu, 610,032 China

**Keywords:** Anti-synthase syndrome, Interstitial lung disease, Anti-alanyl tRNA synthetase, SARS-Cov-2 infection, Autoimmune disease

## Abstract

**Background:**

Anti-synthetase syndrome (AS) is a rare autoimmune idiopathic inflammatory myopathy (IIM) with diverse manifestations, including arthritis, interstitial lung disease (ILD), Raynaud’s phenomenon, unexplained persistent fever, and mechanic’s hands.

**Case presentation:**

We present the case of a 72-year-old woman, previously healthy, who was admitted to our hospital for treatment of cough and rapid breathing. The patient had elevated white blood cells and C-reactive protein, and tested negative for severe acute respiratory syndrome coronavirus 2 (SARS-Cov-2). She was initially diagnosed with community-acquired pneumonia and received tamoxifen for anti-infection treatment, but her dystonia worsened. She eventually required non-invasive ventilator support, tested positive for SARS-Cov-2 again, and started antiviral therapy, corticosteroids to reduce alveolar effusion, anticoagulation, and other treatments. However, her condition continued to deteriorate, with the lowest oxygenation index reaching only 80mmHg. Ultimately, she underwent tracheal intubation and mechanical ventilation. Chest CT revealed rapid progressive interstitial changes in her lungs, and her hands showed noticeable fraternization changes. At this point, we suspected that the novel coronavirus infection might be associated with autoimmune diseases. The patient’s autoimmune antibody spectrum showed positive results for anti-recombinant RO-52 antibody and myositis-specific antibody anti-alanyl tRNA synthetase (anti-PL-12). The patient was treated with dexamethasone sodium phosphate for anti-inflammatory and anti-fibrotic effects. After successful extubation, the patient was discharged with only oral prednisone tablets at a dose of 30 mg.

**Conclusions:**

This case presents an early diagnosis and successful treatment of anti-synthetase syndrome combined with SARS-Cov-2 infection, emphasizing the importance of comprehensive physical examination. Additionally, it highlights the rapid progression of interstitial lung disease under SARS-Cov-2 infection, which is often difficult to distinguish on imaging. In cases where treatment for SARS-Cov-2 infection is ineffective, early screening for autoimmune diseases is recommended. As there is currently no standardized method for treating AS-ILD, the successful treatment of this case provides a reference for clinical research on anti-synthetase syndrome in the later stage.

**Supplementary Information:**

The online version contains supplementary material available at 10.1186/s12890-024-02966-2.

## Introduction

Anti-synthetase syndrome (AS) is an autoimmune disorder characterized by the presence of autoantibodies against amino-acyl-transfer RNA synthase. Interstitial lung disease (ILD) is the leading cause of morbidity and mortality in patients with AS. Anti-PL-12 is the third myositis-specific autoantibody discovered by Bunny et al. in 1986 [1]. In previous studies, patients positive for anti-PL-12 had a lower probability and prevalence of muscle involvement, manipulator, and Maynard phenomenon [2], and ILD was the most common form of lung involvement [3]. Currently, evidence regarding the HRCT patterns of AS-related ILD mainly relies on retrospective case series and case reports. The radiological features of AS are typically nonspecific, and the differential diagnosis of ILD is broad, requiring differentiation from other primary causes, including idiopathic and secondary causes. To achieve a definitive diagnosis, a multidisciplinary evaluation of clinical, serological, and radiological manifestations should be considered for each patient [4]. Pulmonary infection caused by severe acute respiratory syndrome coronavirus-2 (SARS-Cov-2) infection can also lead to multiple ground-glass shadow-like pulmonary interstitial changes. Therefore, ILD caused by AS is often hidden when the two are combined. Although there is currently no standardized method for treating AS-ILD, previous retrospective studies have shown that most AS-ILD cases have a positive response to steroid treatment [5], whether or not immunosuppressive agents or anti-fibrotic drugs such as pirfenidone and nintedanib are used. They mainly slow the progression of the disease by interfering with epithelial-mesenchymal transition and several other processes [6]. SARS-Cov-2 infection can present as rapidly progressing ILD. Failure to conduct careful physical examinations or early consideration of immune-related illnesses can lead to misdiagnosis and delay in treatment. This case provides a new approach for clinical physicians, both in the early diagnosis and treatment process.

## Case presentation

**Fig. 1:**
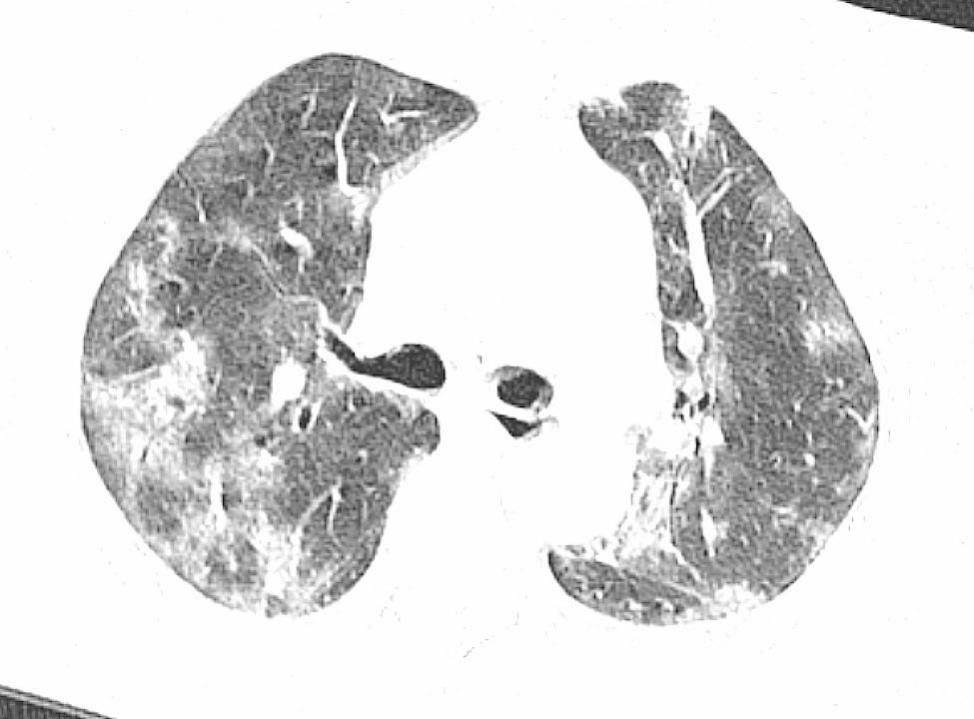
Both lungs scattered in multiple flaky ground glass shadows, mostly subpleura

**Fig. 2:**
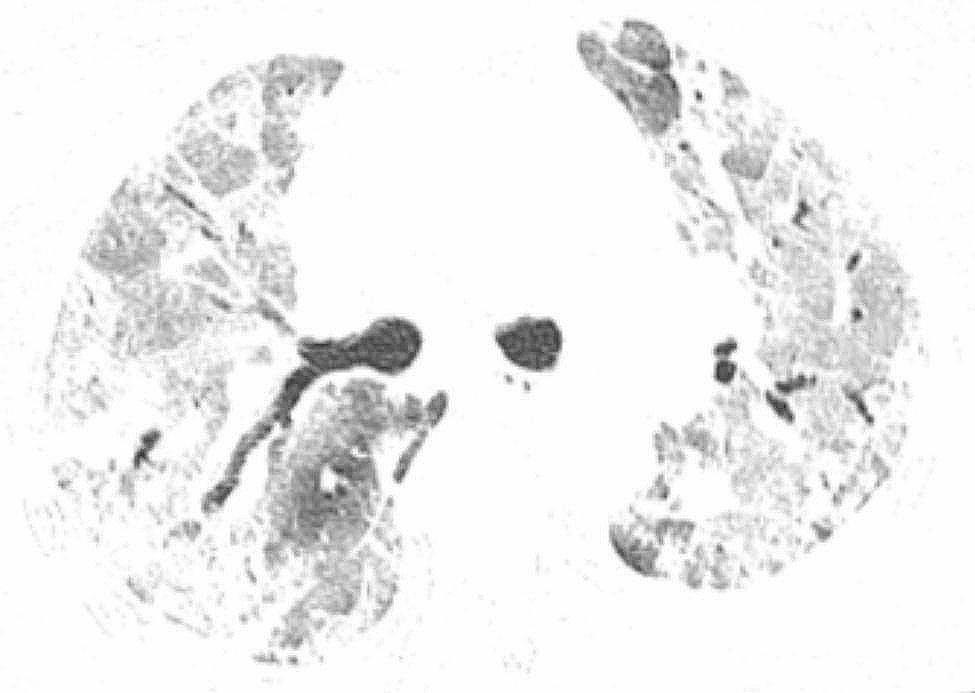
Diffuse ground glass shadows and solid shadows of both lungs

**Fig. 3:**
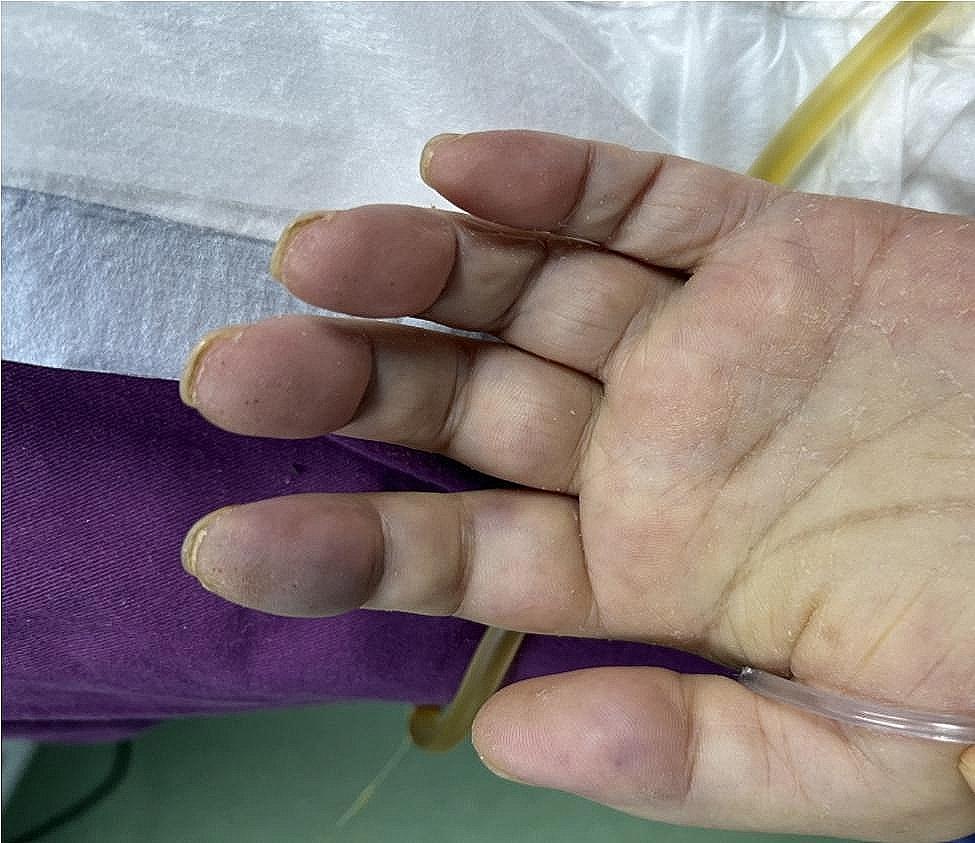
Hyperkeratosis is seen on the lateral tip of the patient’s hand

**Fig. 4:**
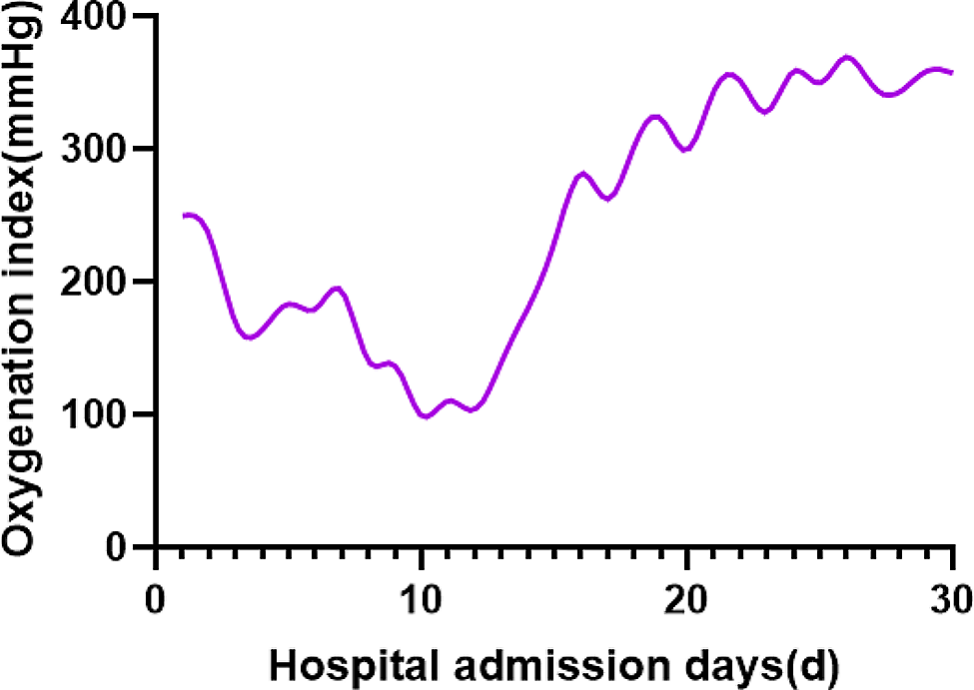
Trend chart of oxygenation index after admission

**Fig. 5:**
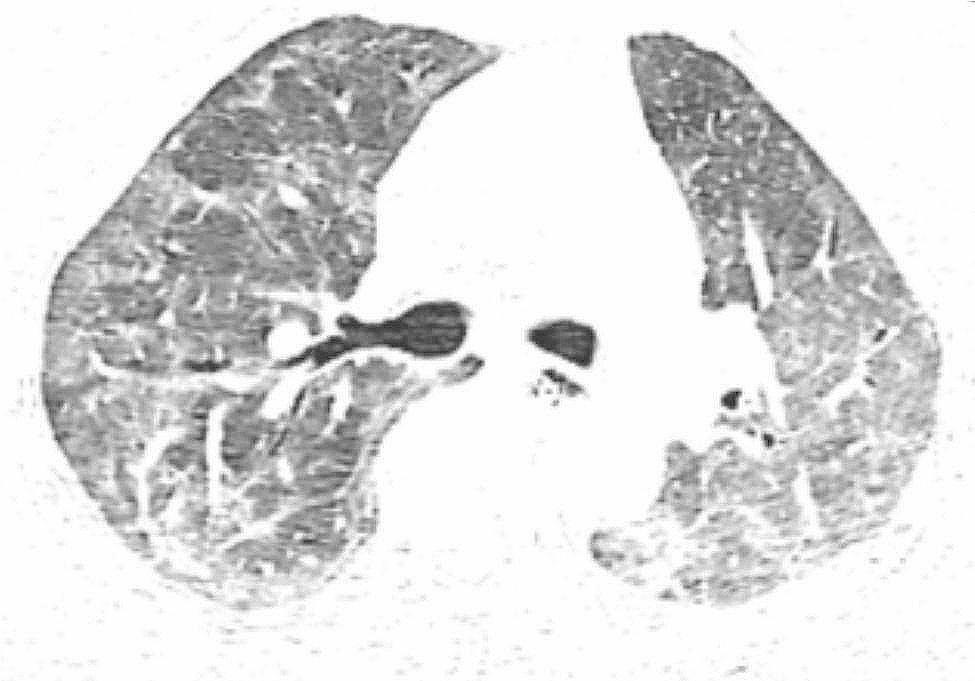
Two lungs scattered in distributed reticulated shadow and ground glass shadow

**Fig. 6:**
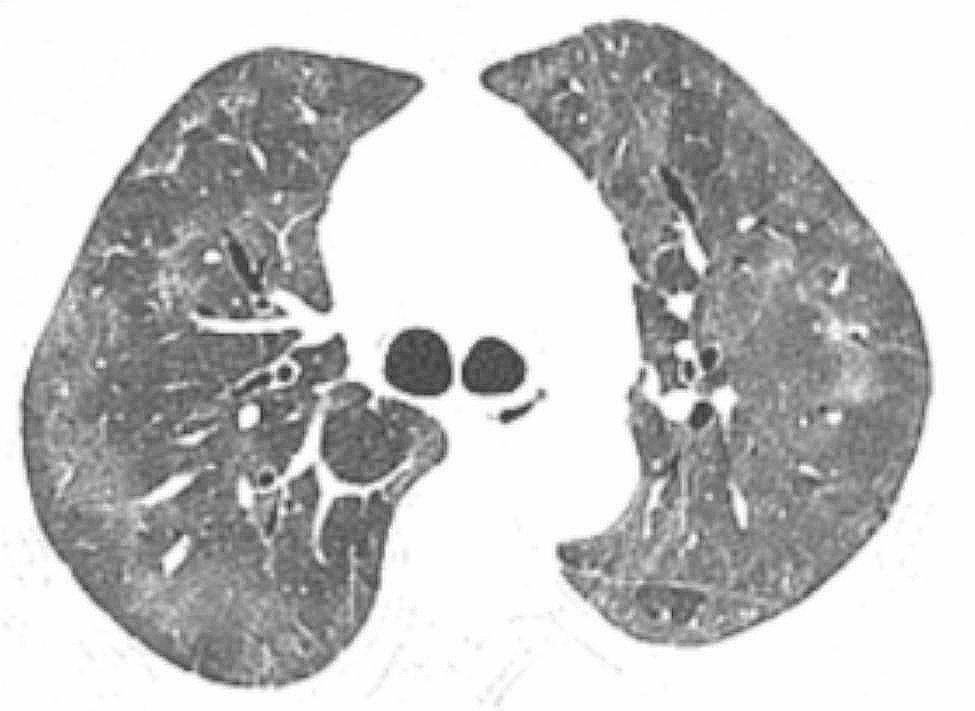
Fibrous changes in basic absorption in both lungs

The patient, a 72-year-old retired nurse, was in good health with no history of alcohol or tobacco use, chronic lung disease, immune disease, family history, and no complaints of muscle pain or weakness. The patient was admitted to our hospital with symptoms of fever, cough, and shortness of breath. Upon examination (Table 1), the patient showed elevated levels of white blood cells and C-reactive protein, and tested negative for SARS-Cov-2 swabs. The patient was diagnosed with community-acquired pneumonia and treated with laroxefin for anti-infection. However, the patient’s dyspnea worsened during treatment. On the third day, the patient required non-invasive ventilator assisted ventilation, and the SARS-Cov-2 swab was tested positive again. The chest CT examination revealed ground glass shadow changes (Fig. 1), and the patient was immediately treated with azivudine 5 mg/day for novel coronavirus infection. Azvudine is a small-molecule antiviral drug taken orally, developed independently in China. It is metabolized into active Azvudine triphosphate in cells and specifically targets the RNA-dependent polymerase of the novel coronavirus, effectively blocking or terminating its replication. Additionally, a daily dose of 40 mg of methylprednisolone sodium succinate for injection is administered to reduce alveolar effusion, along with anticoagulant therapy of 4000IU/day of Enoxaparin sodium. However, the patient’s condition continued to worsen. While using a non-invasive ventilator for assistance, the patient’s oxygen concentration remained at 100%, but their oxygen saturation was still only around 70%, and the lowest oxygenation index was only 80. On the 10th day, the patient was moved to the intensive care unit following endotracheal intubation. A re-evaluation of the chest CT scan revealed extensive interstitial fibrosis changes in both lungs (Fig. 2). We continued the treatment with methylprednisolone sodium succinate for injection at a dosage of 40 mg/day for its anti-inflammatory effects, enoxaparin sodium at a dosage of 4000IU/day for anticoagulation, along with prone positioning, imipenem and cilastatin sodium for anti-sensitivity combined with voriconazole for anti-infection, and compound sulfamethoxazole for the prevention of pneumocystis infection. To determine the cause, we recommended a lung tissue biopsy to the patient’s family, but they declined. After a thorough physical examination, we discovered that the patient was unable to participate in the muscle strength assessment due to sedation and analgesia, and no rash was observed on the entire body. However, we noticed significant roughness and thickening of the skin on the patient’s fingers, along with evident keratinization changes (Fig. 3). At this point, we began to suspect a potential combination of novel coronavirus infection and autoimmune diseases. We conducted auxiliary autoimmune-related tests (Table 2), and the results of the patient’s autoimmune antibody spectrum revealed positive antinuclear antibody (ANA) and anti-recombinant RO-52 antibody (+++). Considering the changes in the skin on the patient’s fingers, we have a strong suspicion of dermatomyositis. Therefore, we promptly conducted a myositis-specific antibody spectrum test, which yielded a positive result for anti-PL-12 using the immunoprecipitation method (Table 3). As per Solomon et al. [7], the patient tested positive for anti-PL-12, and the patient’s chest CT scan indicated interstitial lung disease, which is a key criterion. This contributes to a secondary diagnosis, and patients can be diagnosed with AS as outlined above. Subsequently, we continued using methylprednisolone sodium succinate for injection at a dosage of 80 mg per day, cyclophosphamide for injection at a dosage of 0.4 g per day, and Nidanib softgel ethanesulfonate at a dosage of 150 mg twice a day for anti-fibrosis therapy, while reducing the anti-infection regimen. The patient’s oxygenation index improved from day 16 of admission (Fig. 4), interstitial fibrosis showed significant improvement based on chest CT review (Fig. 5), and the tracheal tube was successfully removed on day 22. At 30 days, the patient only required intermittent nasal catheter oxygen (2 L/min), and we discontinued methylprednisolone sodium succinate, switching to oral prednisone tablets at a dosage of 30 mg per day. The patient was successfully discharged. After discharge, the patient reduced the prednisone dosage by 5 mg per week, and discontinued the prednisone after 6 weeks. A follow-up chest CT showed that the fibrosis in both lungs had been largely absorbed (Fig. 6). Finally, we followed up with the patient, who no longer required oxygen therapy, resumed their normal daily activities, and was able to engage in a small amount of physical activity.

**Table 1:** Laboratory results on admission

Laboratory index	Result	Reference range
White blood cell count (WBC)	13.37 × 10^9^/L	(3.5–9.5)×10^9^/L
Hemoglobin	125 g/L	(115–150)g/L
Red blood cell count (RBC)	4.18 × 10^12^/L	(3.81–5.1)×10^12^/L
Platelets	264 × 10^9^/L	(100–300)×10^9^/L
C- reactive protein (CRP)	65.74 mg/L	(0–5)mg/L
Serum calcium	2.15mmol/L	(2.11–2.52)mmol/L
Serum urea	3.41mmol/L	(3.1–8.8)mmol/L
Serum creatinine	68.1umol/L	(41–81)umol/L
Total protein	79 g/L	(65–85)g/L
Serum albumin	40.2 g/L	(40–55)g/L
Alanine aminotransferase (ALT)	16U/L	(7–40)U/L
Aspartate aminotransferase (AST)	28U/L	(13–35)U/L
Alkaline phosphatase (ALP)	81U/L	(50–135)U/L
creatine kinase (CK)	88U/L	(40–200)U/L
Serum lactate dehydrogenase (LDH)	297U/L	(120–250)U/L

**Table 2:** Results of autoimmune related auxiliary tests

Autoantibody	Result
Anti-pm-scl antibody	Negative
Anti-mitochondrial M2 antibody	Suspicious positive(±)
Anti-ribosome P protein antibody	Negative
Anti-histone antibodies	Negative
Anti-nucleosome antibody	Negative
Anti-cyclin antibody	Negative
Anti-centromere B antibody	Negative
Anti-jo-1 antibody	Negative
Anti-scl-70 antibody	Negative
Anti-SSB antibody	Negative
Anti-recombinant RO-52 antibody	positive(+++)
Anti-SSA antibody	Negative
Anti-Sm antibody	Negative
Anti-U1RNP antibody	Negative
Anti-double-stranded DNA antibody	Negative
Anti-cyclic citrulline peptide antibody	Negative
Rheumatoid factor	Negative
Anti-neutrophil cytoplasmic antibody	Negative
Anti-glomerular basement membrane antibody	Negative
Antinuclear antibody	positive(+)

**Table 3:** myositis antibody spectrum

Antibody name	Result
anti-7-2-ribonucleoprotein antibody	Negative
anti-Ku antibody	Negative
anti-Mi-2 antibody	Negative
anti-PM-SCL100 antibody	Negative
anti-PM-SCL75 antibody	Negative
anti-RNA polymerase III antibody	Negative
anti-Ro52 antibody	positive
anti-U1-RNP antibody	Negative
anti-phenylalanyl tRNA synthetase antibody	Negative
anti-alanyl tRNA synthetase antibody	positive
anti-Sjögren’s syndrome B antibody	Negative
anti-glycyl tRNA synthetase antibody	Negative
anti-nuclear matrix protein 2 antibody	Negative
anti-melanoma differentiation-associated gene 5 antibody	Negative
anti-tyrosyl tRNA synthetase antibody	Negative
anti-seryl tRNA synthetase antibody	Negative
anti-threonyl tRNA synthetase antibody	Negative
anti-topoisomerase 1 antibody	Negative
anti-mitochondrial-M2 antibody	Negative
anti-small ubiquitin-like modifier-activating enzyme antibody	Negative
anti-signal recognition particle antibody	Negative
anti-isoleucyl tRNA synthetase antibody	Negative
anti-fibrillarin antibody	Negative
anti-transcription intermediary factor 1-γ antibody	Negative
anti-glutamyl tRNA synthetase antibody	Negative
anti-Ro60 antibody	Negative
anti-centromere antibody	Negative

## Discussion

AS is a rare clinical disease with an unclear prevalence, characterized by the ILD, arthritis, and myositis triad. In addition to the typical triad, other features include Raynaud’s phenomenon, unexplained fever, and mechanic’s hands. These additional symptoms are present in only 40% of patients [8]. The main challenge in diagnosing AS lies not only in its rarity but also in the fact that only 20% of patients will exhibit these triads. In this case, the main manifestation is rapidly progressing ILD, which is also a major presentation in critically ill COVID-19 patients. Previous studies have shown that SARS-CoV-2 infection itself can induce the expression of various fibrotic factors [9], and COVID-19-related acute exacerbation of ILD has a worse prognosis than non-COVID-19 acute exacerbation [10]. Pulmonary fibrosis is not only a disease risk, but also a possible complication of COVID-19. Additionally, in high-resolution CT imaging, both may exhibit similar radiological features, such as traction bronchiectasis, ground-glass opacities, and reticular changes. The previous autoimmune abnormalities are independent risk factors for COVID-19 patients, or whether SARS-CoV-2 infection can lead to autoimmune abnormalities in patients, these questions currently have no definite answers [11]. Due to the rarity of autoimmune diseases like AS and the ease of missing its diagnosis even under normal circumstances, clinicians may be biased towards diagnosing COVID-19 during the pandemic, especially when SARS-Cov-2 swabs are positive. As a result, the possibility of connective tissue-associated interstitial lung disease may be overlooked when the two conditions are combined. Previous studies [12] have reported a case with radiological findings of interstitial pneumonia. Although the result for SARS-CoV-2 was negative, clinical physicians still treated the patient as if it were COVID-19. After further screening, it was eventually confirmed to be AS. Fortunately, the patient’s pulmonary interstitial changes were far less severe than in this case, so even with a delay in the initial diagnosis, a favorable outcome was still achieved. Therefore, a thorough physical examination is crucial, and if abnormal autoimmune antibody spectra are detected, AS should be highly suspected. It is important to promptly enhance the detection of myositis antibody spectrum. Multiple factors interact and often result in delayed diagnosis of AS. In terms of treatment, the focus is on reducing inflammation and the production of autoantibodies. Currently, there is no standardized approach to treating AS-ILD due to the lack of results from randomized controlled trials of various drugs. Most protocols are derived from relevant literature, including AS cohorts and case reports of refractory diseases. ILD is a significant predictor of mortality and prognosis. It is typically refractory, and its presence or absence will greatly impact the treatment and prognosis of patients, and drive the advancement of immunosuppressive therapy. High dose corticosteroids form the foundation of anti-synthetase syndrome treatment. However, treatment is not standardized and we should consider both the affected organ systems, as well as the speed and severity of organ damage. No controlled trials have been conducted to determine the superiority of corticosteroids over other immunosuppressants in the initial treatment of active disease [13]. However, because of the extended length of corticosteroid treatment and the potential for corticosteroid resistance [14], we frequently initiate immunosuppressive medications to decrease relapse while slowly tapering the steroid dosage. Cyclophosphamide is often prescribed for severe cases of AS-ILD. Research has indicated that even though the dosages of cyclophosphamide and combined immunosuppressants may differ, approximately 71% of patients experience improved lung function [15]. In addition, nidanib is an intracellular inhibitor that targets multiple tyrosine kinases. Several studies have demonstrated that a dosage of 150 mg of nidanib taken twice daily can help reduce the decline in pulmonary function and FVC in patients with idiopathic pulmonary fibrosis [16, 17]. However, there is a lack of studies on the impact of nidanib on AS-ILD treatment. Nevertheless, in this particular case, we observed a positive effect on pulmonary fibrosis, which could be validated through further clinical studies in the future. In summary, this case serves as a reminder for clinicians to consider various indicators when diagnosing ILD in order to avoid missed or incorrect diagnoses. Compared to previously reported cases [18] of COVID-19 combined with AS, the interstitial changes in this patient are more severe. Currently, there are no specific standards for the dosage and duration of treatment with steroids and immunosuppressants for AS. The successful treatment of this case provides a direction for future research on AS-ILD treatment.

### Electronic supplementary material

Below is the link to the electronic supplementary material.

Supplementary Material 1

## Data Availability

No datasets were generated or analysed during the current study.
